# Implementing work-related Mental health guidelines in general PRacticE (IMPRovE): a protocol for a hybrid III parallel cluster randomised controlled trial

**DOI:** 10.1186/s13012-021-01146-8

**Published:** 2021-08-04

**Authors:** Danielle Mazza, Samantha Chakraborty, Vera Camões-Costa, Justin Kenardy, Bianca Brijnath, Duncan Mortimer, Joanne Enticott, Michael Kidd, Lyndal Trevena, Sharon Reid, Alex Collie

**Affiliations:** 1 Department of General Practice, Monash University, Melbourne, Australia; 2 University of Queensland, Brisbane, Australia; 3 National Ageing Research Institute, Parkville, Australia; 4 School of Allied Health, Curtin University, Perth, Australia; 5 Centre for Health Economics, Monash Business School, Monash University, Melbourne, Australia; 6 Monash Centre for Health Research and Implementation, Monash University, Melbourne, Australia; 7 College of Health and Medicine, The Australian National University, Canberra, Australia; 8 Sydney School of Public Health, Faculty of Medicine and Health, The University of Sydney, Camperdown, Australia; 9 Insurance Work and Health Group, Monash University, Melbourne, Australia

**Keywords:** Guideline implementation, General practice, Hybrid III trial, Mental health, Work, Integrated Knowledge Translation

## Abstract

**Background:**

The Clinical Guideline for the Diagnosis and Management of Work-related Mental Health Conditions in General Practice (the Guideline) was published in 2019. The objective of this trial is to implement the Guideline in general practice.

**Trial design:**

Implementing work-related Mental health conditions in general PRacticE is a hybrid III, parallel cluster randomised controlled trial undertaken in Australia. Its primary aim is to assess the effectiveness of a complex intervention on the implementation of the Guideline in general practice. Secondary aims are to assess patient health and work outcomes, to evaluate the cost-effectiveness of the trial, and to develop a plan for sustainability.

**Methods:**

A total of 86 GP clusters will be randomly allocated either to the intervention arm, where they will receive a complex intervention comprising academic detailing, enrolment in a community of practice and resources, or to the control arm, where they will not receive the intervention. GP guideline concordance will be assessed at baseline and 9 months using virtual simulated patient scenarios. Patients who meet the eligibility criteria (>18years, employed, and receiving care from a participating GP for a suspected or confirmed work-related mental health condition) will be invited to complete surveys about their health and work participation and provide access to their health service use data. Data on health service use and work participation compensation claim data will be combined with measures of guideline concordance and patient outcomes to inform an economic evaluation. A realist evaluation will be conducted to inform the development of a plan for sustainability.

**Results:**

We anticipate that GPs who receive the intervention will have higher guideline concordance than GPs in the control group. We also anticipate that higher concordance will translate to better health and return-to-work outcomes for patients, as well as cost-savings to society.

**Conclusions:**

The trial builds on a body of work defining the role of GPs in compensable injury, exploring their concerns, and developing evidence-based guidelines to address them. Implementation of these guidelines has the potential to deliver improvements in GP care, patient health, and return-to-work outcomes.

**Trial registration:**

ACTRN12620001163998, November 2020

**Supplementary Information:**

The online version contains supplementary material available at 10.1186/s13012-021-01146-8.

Contributions to the literature * An intervention that implements an evidence-based clinical guideline equipping GPs with recommendations, resources, and networks to improve GP care.

- Improvements in GP care that enhance patient recovery, work productivity, and reduce health service utilisation and compensation claims.
- A demonstration of how researchers and policy makers can work with GPs to facilitate the translation of research into clinical practice.
- Rigorous evaluation processes and measurable feasibility and economic value mean more compensation partners can nationally roll out the intervention.

## Background

Mental health conditions that have arisen as a result of work factors (henceforth referred to as work-related mental health conditions) are one of the most challenging injuries to manage [1, 2]. These conditions can result in some of the most expensive and complex claims and afflicted patients can take three times longer to return to work compared with the median time for all claims [3].

GPs play a key role in the diagnosis, treatment, and care of patients with work-related injuries. They are perceived as one of the most trusted sources of advice for patients, considered by patients and other health professionals to be the primary coordinator of patient care, and are often tasked with advocating to employers on the patient’s behalf [2, 4, 5]. Yet, many GPs, internationally, describe substantial challenges in providing this care, particularly where the injury involves a mental health condition [4–9]. For instance, GPs describe difficulty diagnosing mental health conditions and determining the work-relatedness of a mental health condition, navigating system complexities, coordinating clinical care, communicating with workplaces, concern about imposing further harm on the patient (for instance concern about the patient facing stigma at work if they are known to have a work-related mental health condition), and a lack of evidence about optimal clinical practice [5, 8, 10, 11]. That similar challenges are described internationally underscores that across the compensable injury landscape, patients with mental health conditions that have arisen from work are seen as difficult to manage, and that GPs are fundamental to brokering improved health and employment outcomes for such patients. The nature of these challenges suggests that they can be overcome through interventions that improve knowledge and offer GPs the opportunity to grow their confidence and skills in providing care.

In 2019, an evidence-based clinical guideline titled *Clinical guideline for the diagnosis and management of work-related mental health conditions in general practice* was published [12]. This guideline is the only GP-focused guideline on diagnosing and managing work-related mental health conditions internationally. It was developed according to the gold-standard for guideline development in Australia—and approved by the National Health and Medical Research Council—and is endorsed by the two peak general practice membership organisations in Australia, the Royal Australian College of General Practitioners and the Australian College of Rural and Remote Medicine. It addresses specific clinical dilemmas about the diagnosis and management of work-related mental health conditions that have been raised by GPs and other clinical and policy stakeholders [11, 13].

Despite the Guideline being available, it is widely recognised that clinicians seldom consult clinical guidelines when making clinical decisions [14]. Instead, they more often develop internalised, tacit protocols that are based on collective reinforcement by peers, previous clinical experiences, and, to a lesser extent, literature or guidelines [14]. To effectively embed guideline-concordant behaviour into practice, an intervention would need to facilitate the development of new collectively reinforced, internalised tacit beliefs that align with the Guideline recommendations and overcome the concerns described by GPs. These requirements align well to the constructs of normalisation process theory: coherence, cognitive participation, collective action, and reflexive monitoring [15]. Therefore, an intervention that operationalizes normalisation process theory and re-enforces the role of peers and opinion leaders [16, 17] may be effective at implementing the Guideline. Whilst these factors ready a clinician for behaviour change, the application, practice, and embedding of new practices occurs over time and stages [18]. As such, a combination of these elements may provide an effective and sustainable model for influencing guideline-concordant behaviour change in GPs.

Two intervention approaches that encompass the principles of normalisation process theory and the influence of peers and opinion leaders are academic detailing and communities of practice. These interventions have some success in changing GP behaviour when offered individually [19–22], but are more effective when offered together [19, 23]. Academic detailing involves peer-to-peer delivery of evidence-based key messages that are tailored to the needs of participating clinicians [20, 21, 23]. Academic detailing has been used regularly to educate clinicians and facilitate evidence-based practice [24].

Additionally, a community of practice involves driving innovation and excellence through sustained interaction over a period of time [22, 25]. Communities of practices have been used successfully in Australia to improve evidence-based care and communication skills in general practice [26]. They provide support for practitioners who can feel isolated in dealing with clinical problems, helping them to create networks and implement new models of care [27]. A third approach, the provision of resources to support guideline implementation, is also more effective when combined with peer-to-peer strategies [28].

**Fig. 1:**
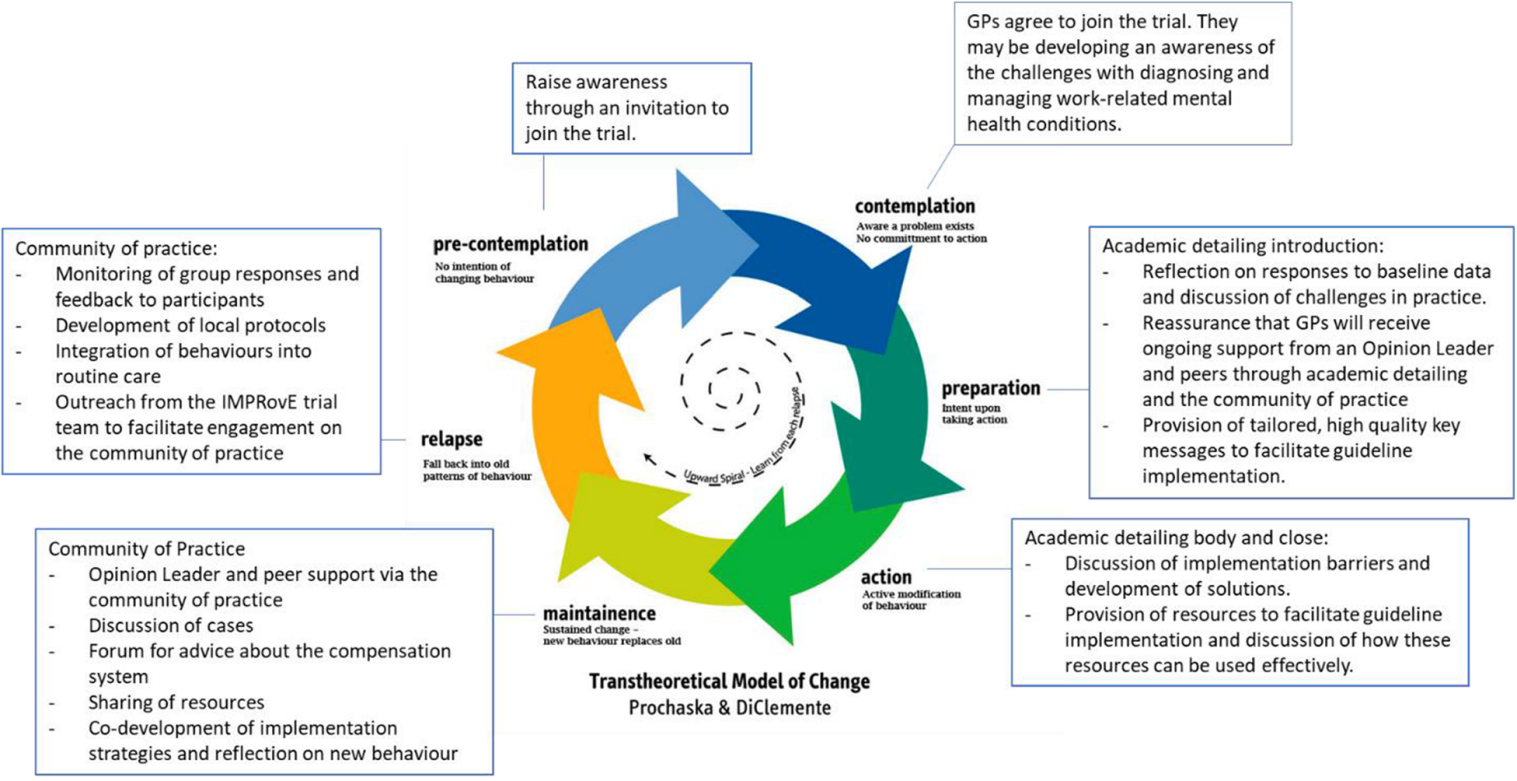
Theoretical basis for the IMPRovE intervention based on Prochaska and DiClemente Transtheoretical Model of Change [18]

Our complex intervention combines these academic detailing a community of practice and the provision of resources and is expected to facilitate guideline implementation by introducing these in a staged manner to facilitate guideline-concordant practice over time (Figure 1). It will do so in the following ways: (a) addressing professional isolation; (b) learning from respected peers; (c) enabling GPs to demonstrate their own leadership by assisting their peers; (d) enabling GPs to develop confidence in their plan of action before consulting with the patient, or stakeholder involved in the patient’s care; and (e) providing this interaction in a flexible environment that caters to the GP’s resource availability (e.g. time, mode of communication).

## Research hypothesis

Our primary hypothesis is that exposure to a complex intervention consisting of academic detailing, and provision of resources and engagement in a digital community of practice will increase the delivery of guideline-concordant care by GPs to patients regarding the diagnosis and management of work-related mental health conditions. We hypothesise that increases in guideline-concordant care will translate into improvements in health status and work productivity, and reductions in health service utilisation.

## Aims

This study aims to assess the effectiveness of a complex intervention involving academic detailing, provision of resources, and engagement in a digital community of practice on the implementation of evidence-based guidelines for the diagnosis and management of work-related mental health conditions in general practice. Our secondary aims are to (a) improve the health and return-to-work outcomes for patients with work-related mental health conditions in this study, (b) evaluate the cost-effectiveness of the guideline implementation strategy, and (c) develop an implementation plan for sustainability.

## Methods

### Design

**Fig. 2:**
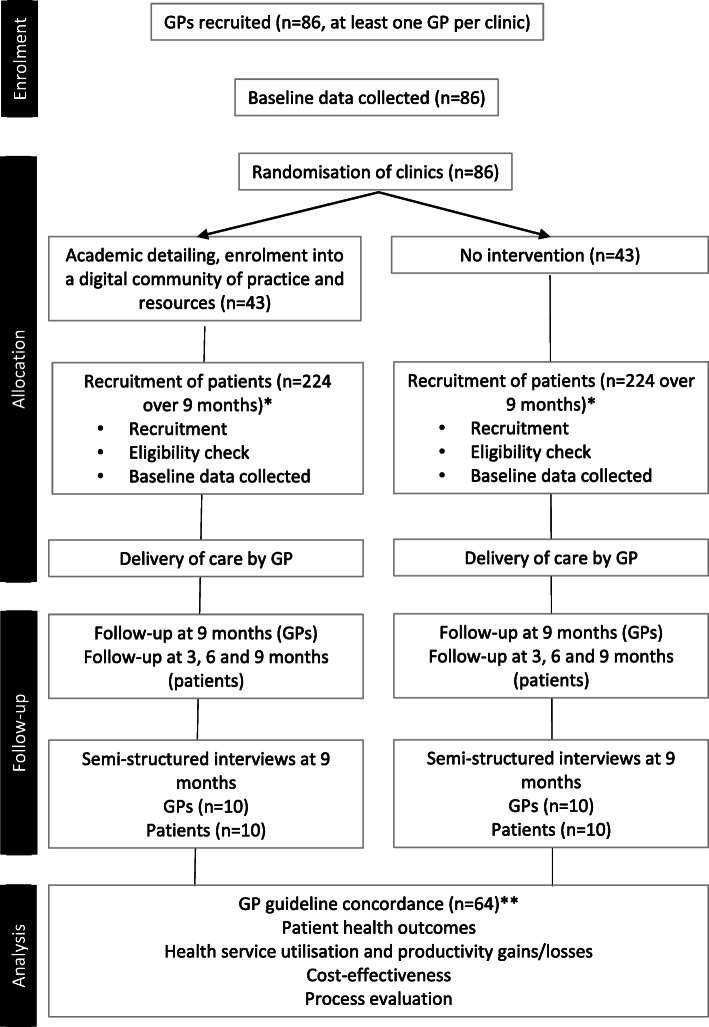
CONSORT flowchart for the IMPRovE trial design and outcomes. *Patient numbers calculated assuming 25% of GPs will drop out of study. **GP guideline concordance calculated assuming 25% of GPs will drop out of the study

Implementing work-related Mental health conditions in general PRacticE (IMPRovE) is a hybrid III parallel cluster randomised controlled trial (RCT) of a complex intervention that involves receipt of academic detailing, enrolment onto a digital community of practice, and access to a library of resources to support guideline implementation. GP clinics (clusters) will be randomised to either intervention or control. A cluster design was chosen to avoid confounding that could arise if participating GPs from a single clinic were allocated to different arms of the trial. Participating GPs in clinics assigned to the intervention arm will receive the intervention. GPs in the control arm will not receive the intervention. Patients will be recruited to each arm (Figure 2).

As a hybrid III trial [29], the primary outcome of the trial will demonstrate the effectiveness of this complex intervention to improve guideline concordance by GPs. The secondary outcomes of the trial will demonstrative the effectiveness of the complex intervention on patient heath status and cost-effectiveness. Lastly, we will review the likelihood of this intervention being sustained and develop a plan for sustainability. This protocol has been reported according to the Template for Intervention Description and Replication (TIDieR) checklist [30] and the CONSORT extension for Cluster Trials [31] (Additional files 1 and 2, respectively).

### Setting

The study will be conducted across all states and territories in Australia. Recruitment of GPs will occur from December 2020 until December 2021 whilst recruitment of patients will occur from February 2021 until September 2022 or the latest date representing nine-months after the final GP provides baseline measures.

### Inclusion and exclusion criteria

#### General practitioners

GPs are eligible to participate if they can recruit 7 to 24 eligible patients over the course of the trial. GPs may consent after their practice has already been randomised; however, we will aim to enrol all GPs before their practice has been notified of their allocation. If a GP works across multiple sites, then we will randomise both sites together.

#### Patients

Patients are eligible if they are 18 years or older, have a confirmed or suspected work-related mental health condition, are receiving care from a participating GP, and are employed at the time of enrolment. Patients who are employed but are not working (e.g. on paid or unpaid leave) and those who have an active workers’ compensation claim are also eligible to participate in the study.

### Participant recruitment

#### General practitioners

GPs meeting the minimum caseload requirements for eligibility will be recruited through regional health authorities (known as primary health networks), GP membership organisations, study partners, and other relevant healthcare organisations using newsletters, website updates, and social media avenues.

#### Patients

Patients will be recruited via the participating clinic through advertisement in the clinic waiting room, advertisement on the participating GP’s desk, or directly through the participating GP, who will identify eligible patients and provide these patients with a printed information pack about the study. The information pack will include an explanatory statement, consent forms, and a reply-paid envelope. In addition to seeking consent to participate in data collection, consent will also be requested from patients to access administrative records of their health service utilisation for the purposes of an economic evaluation. Patients who wish to participate in the study will be invited to contact the IMPRovE project team for further information or to return their completed consent forms directly to the team using the reply-paid envelope.

### Sample size

A sample size of 86 clinics (clusters), with a minimum of one GP per cluster and assuming a GP dropout rate of 25%, has >80% power to detect a medium-large effect size of Cohen’s d of 0.69 (assuming a GP-level outcome score change of 1.2 on a scale with score range of 0 to 9 and a standard deviation of 1.75 as identified in a pilot study of the primary outcome tool; correlation between baseline and follow-up measurements of 0.3, and two-sided significance of 0.05).

We estimate that each GP cluster will recruit a minimum of 7 and a maximum of 24 patients (the cut-off to ensure that the patient sample is not skewed by a high-recruiting cluster) over the study period. Based on our analysis of high-caseload GPs [32], we estimate that there will be approximately 22 eligible patients/year/GP. Accounting for a 66% estimated rate of refusal to participate as observed in another study that recruited participants with mental health conditions in the Australian general practice setting [33], we estimate that at least 7 eligible patients will be successfully recruited by each participating GP in a 9-month period.

We anticipate that approximately 448 patients who are recruited within the 9 months (224 from control clinics and 224 test clinics; 7 per clinic/cluster) will have 3-month data. These patient numbers assume up to 25% of the 86 recruited GPs will drop out of the study. Using data from these patients, we will have 90% power to detect medium effects (Cohen’s d = 0.5) in secondary outcome measures between two groups (test/control) at 3 months. Calculations assume an alpha of 0.01, ICC of 0.03, and correlation of 0.5 between individual scores. Calculations were made using Cluster statistical software [34].

### Randomisation

A statistician who will not be involved in recruitment and will be blinded to the identity of participating GPs will allocate GP clusters to either the intervention or the control arm. Randomisation will be performed as stratified randomisation using a minimization procedure. Stratification factors are state, clinic size (GP equivalent full time >5 (yes/no)), and location (urban vs regional/rural). We will randomise clinics in blocks of two, or every 2 weeks, whichever occurs first.

### Intervention

#### Intervention components

The intervention components have been co-designed by the research team, an Intervention Advisory Group comprising policy and practice representatives, and a key non-government mental health organisation *Beyond Blue* and piloted by a GP reference group. A steering group comprising key practice and policy representatives from across Australia reviewed the intervention design and provided advice to enhance its usability and sustainability. This approach was used to ensure that GPs receive an intervention that is relevant to their clinical practice as well as relevant to policy contexts.

Academic detailing will be provided in a single online session using video-conferencing. The session will be led by a GP opinion leader along with a professional academic detailer. GP opinion leaders are GPs having been nominated by compensation scheme partners as these GPs are well versed in the complexities of the workers’ compensation system but are not representatives of the compensation schemes themselves. Each GP opinion leader will be supported by an experienced academic detailer who has expertise in providing educational outreach programs to GPs. GPs from clusters in the intervention arm of the trial will be detailed on how to improve care for patients with work-related mental health conditions in accordance with the guideline. The format of each session will include (1) introduction to the guideline and the provision of statistics about work-related mental health conditions; (2) discussion of the key challenges that are faced by attending GPs; and (3) discussion of a case study with advice and instruction on how to implement guideline-concordant care. During the academic detailing session, opinion leaders will invite GPs to join the digital community of practice, enrol them as members of this community, and provide a demonstration on how to navigate and use the digital community of practice platform. Opinion leaders will keep a log of the key challenges and issues reported by GPs at each detailing session and this log will be used to inform the content of the community of practice and contribute to the process evaluation. To ensure fidelity of the academic detailing, all academic detailing sessions will be recorded. A random selection of 20% of sessions will then be assessed against a pre-determined checklist, adapted from Carroll et al. [35] for academic detailing sessions, and a fidelity score will be reported. In addition, a qualitative and quantitative fidelity assessment will be used to assess the fidelity of the first five academic detailing sessions and provide feedback to GP opinion leaders on the delivery of these sessions.

The digital community of practice is an online platform that will showcase the guideline recommendations and provides avenues to discuss and support guideline implementation strategies. A central component of the platform will be a discussion forum. In addition, the platform will offer a range of regular content in the form of webinars, case discussions, and news. A content roster and engagement protocol will also be used to facilitate ongoing engagement. The content roster will be reviewed quarterly to adjust for ongoing developments in published educational materials or other resources, and in response to GP engagement levels over the preceding quarter. Once GPs are enrolled into the digital community of practice, they will continue to have access to the community of practice for the duration of the trial (until September 2022).

Participating GPs will be provided with resources including the full guideline, flowchart of guideline recommendations, and a checklist of clinical indicators that was created by the expert Guideline Development Group during development of the guideline. They will also be supplied with resources from the compensation partners and *Beyond Blue*, a prominent non-government mental health organisation in Australia*.*

#### Control group

Participating GPs in clinics assigned to the control group will receive no support for guideline implementation. At the conclusion of the trial, GPs in the control group will be invited to attend an academic detailing session and engage in selected activities from the community of practice.

### Outcomes

#### Primary outcome

The primary outcome is concordance with guideline recommendations by participating GPs. Guideline concordance will be assessed at baseline and 9 months later using virtual simulated patient scenarios. At each data collection point, GPs will be presented with three virtual scenarios of varying levels of complexity and patient circumstance. The three virtual scenarios for each GP at each data collection point will be randomly selected from a set of eighteen virtual scenarios, controlling for learning effects and order effects, and balancing rigorous measurement with participation burden. We based the development of these scenarios on 20 interviews with patients who have lived experiences of work-related mental health conditions to ensure that virtual scenarios reflect the range of patient characteristics that a GP would see.

This approach has been used previously to evaluate practice and as an educational tool in compensable injury research with GPs in Europe [36] and with physiotherapists in Australia [37]. The virtual scenarios are approximately 15 min duration each and focussed on typical presentations to GPs by patients with work-related mental health conditions. Each virtual scenario includes a short video of a simulated patient who describes their symptoms, circumstances and context. Participating GPs are then prompted to respond to questions about how they would assess, diagnose, and manage the patient in the virtual scenario.

Guideline-concordant responses to the scenarios will be measured using a tool that reflects the checklist of indicators that have been included in the Guideline. Responding to each set of three virtual scenarios can result in a best score of 9 or a worst score of 0. The tool was pilot tested with three groups: (1) GPs who are experts in the Guideline, (2) GPs who are not familiar with the Guideline, and (3) medical and research students who have no clinical general practice expertise. The tool was found to have a scale reliability score of Cronbach’s *α*=0.74.

#### Secondary outcomes

Secondary outcomes will capture the effect of the intervention on patient health status and work participation. Patient health status will be measured using the short-form 36-item version 2 (SF-36 v2) [38] and the Depression and Anxiety Stress Scale-21 item (DASS-21) [39]. Work participation will be measured using the return to work items within the Australian National Return To Work Survey [40].

The SF-36 v2 provides measures for mental health symptoms and function, as well as measures of physical function [38]. Since patients with work-related mental health conditions are likely to experience both psychological and physical ill health, the SF-36 v2 is an appropriate measure to assess health status [41]. Due to heterogeneity in the mental health conditions experienced by participating patients, it is necessary to use a broad measure of health that is capable of measuring changes in psychological health. The SF-36 v2 Mental Component Score and Mental Health Domain Score are strongly correlated with measures for depression, anxiety, post-traumatic stress disorder, and substance use [42], which are common work-related mental health conditions. The SF-36 v2 is also sensitive to change following intervention.

The DASS-21 assesses the presence and severity of the core symptoms of depression, anxiety, and stress [39]. Accordingly, the DASS-21 allows not only a way to measure the severity of a patient’s symptoms but a means by which a patient’s response to treatment can also be measured. The National Return To Work survey has been used by the Australian government for over 18 years to assess level of work participation for people who are working and those who are not [40].

Patient endpoints are at the time of their initial appointment with the GP (T0, baseline) and every 3 months after that for 9 months. Patients will be provided paper or electronic links to relevant surveys (including specific measures of psychological disorder) or they will be able to provide their responses via a structured telephone interview, as per their preference. Data for both GP and patient outcomes will be collated electronically using REDCap [43].

### Economic evaluation

The economic evaluation will take a societal perspective in quantifying the additional costs (savings) and health gains associated with the intervention within the trial period. The primary outcome for the economic evaluation will be GP concordance with guideline recommendations. The secondary outcome for the economic evaluation will be quality-adjusted life-years (QALYs) in participating patients; calculated based on SF-36 v2 data at each time-point [44] and using an area-under-the-curve approach.

The cost analysis will capture direct costs of the intervention and control conditions (based on project records and fidelity data), medical costs during the trial period, and productivity gains during the trial period. Medical costs will be estimated based on patient-level administrative data from the Medicare Benefits Scheme (MBS), from Pharmaceutical Benefits Scheme (PBS), and from workers’ compensation schemes (for those patients who submit a claim and provide consent for data access), plus patient self-report of health service use funded by other payers (e.g. hospitalisations and allied health care). Productivity gains will be estimated based on patient-level data regarding return to work from the National Return To Work Survey items. Results of the economic evaluation will be expressed as cost per point improvement in concordance with the Guideline, and cost per QALY gained.

### Realist evaluation

A realist evaluation of the intervention will be conducted using three methods: a knowledge, attitudes, and practice questionnaire provided to GPs at baseline and 9 months later, interviews with a random sample of 20 GPs and 20 participants upon completion of the intervention, and qualitative analysis of the social interactions on the community of practice. All decisions, tailoring and modifications to the structure, format, content, or design of the community of practice will also be documented in a log book.

### Planning for sustainability

A stakeholder workshop will be held to identify how the intervention can be sustained following completion of the trial. Outcome data from the intervention, economic evaluation, and process evaluation will be presented for consideration and reflection by participants.

### Blinding

Given that the intervention involves active engagement with GPs, it is inevitable that GPs will become aware of their group allocation as the study progresses. Patients, however, will not be informed about whether their GP clinic has received the intervention. Clinical trial coordinators will be blinded to the group allocation during quantitative and qualitative data collection; however, it is possible that they will be able to identify a GP’s allocation by the content of their conversations. The study project officer and research assistant will be involved in coordinating and delivering the intervention and will, therefore, not be blinded to group allocation. All GP and patient data will be de-identified by the research assistant before analysis for the primary outcome.

### Statistical methods

#### Primary analysis

The main analysis examining the primary outcome (proportion of guideline concordance) will be intention to treat using multilevel regression models (linear or Poisson regression, as appropriate), with time-point and intervention status as fixed effects. Clusters will be examined as a random effect for inclusion into the model. Covariates will include the stratification factors, which are state, clinic size (GP equivalent full time >5 (yes/no)), and location (urban vs regional/rural).

#### Secondary analysis

*Patient analyses*: Using a similar mixed model approach as in the main analyses, changes in patient health status (using the SF-36 v2), and work participation (using relevant measures of the National Return To Work Survey) will be measured by comparing the scores on patient surveys from baseline to 3, 6, and 9 months following their initial GP consultation.

*Adjusted analyses*: The main analysis and subgroup analyses above include adjustment for the stratification factors, which are state, clinic size (GP equivalent full time >5 (yes/no)), and location (urban vs regional/rural), additional mental health training.

*Missing data*: As the GP and patient-level data are longitudinal samples, a missing data analysis will be done on each longitudinal data set and investigations made for predictors of missingness. These investigations will examine the effect of key demographic and other factors particularly on the presence of missing outcomes. If outcome data is found to not be missing at random, then multiple imputation will be applied using the predictors for missingness.

### Data monitoring

A data monitoring committee will meet quarterly to review study data during the period of recruitment and intervention, and interim analysis will be supplied to the Executive Team at scheduled occasions and on request and in confidence.

### Harms

We do not anticipate any harms to GP or patient participants as a result of participating in this study.

## Discussion

The challenges described by some GPs when managing work-related mental health conditions reflect gaps in knowledge as well as the absence of pragmatic effective strategies to implement Guideline recommendations and a likely lack of confidence in implementing these strategies [4, 11]. The IMPRovE intervention will address these challenges using a complex intervention that comprises academic detailing, a community of practice, and the provision of resources to improve Guideline-concordance amongst participating GPs.

Firstly, tailored academic detailing will be provided to each GP cluster in the intervention. This session will be led by a GP opinion leader, who will introduce the Guideline, identify evidence-based practice gaps in current clinical practice, and provide GP participants with practical and tailored strategies during the academic detailing session. This phase of the intervention will raise awareness of the issue amongst participants, highlight areas of behaviour change in their own practice, and create an initial sense of enthusiasm regarding implementing the Guideline recommendations. Secondly, the digital community of practice will provide GPs with opportunities to increase their learning; receive advice about other clinical dilemmas that were not addressed in the initial academic detailing session, or test and adjust their own practices; and assist their colleagues to develop implementation strategies for a range of issues related to mental health conditions that have arisen as a result of work. Engagement with the GP opinion leader will continue on the community of practice platform, thus re-enforcing the credibility of discussions and learnings on the community of practice. Finally, during the academic detailing session, GP participants will receive a suite of resources that can assist them to implement the Guideline, along with advice about how to use these resources (e.g. to monitor patient improvement), and a more comprehensive resource library will be offered via the community of practice.

The IMPRovE intervention has been constructed in response to the self-reported barriers that sometimes prevent GPs from providing optimal care for people with work-related mental health conditions [11], as well as theoretical principles of normalisation process theory [15]. As the IMPRovE intervention involves both peers and opinion leaders, they offer participating GPs the peer-to-peer social influences that are well regarded by clinicians when making choices about their clinical practice [17]. The flexible nature of the academic detailing session enables the GP opinion leader to adapt a session so that it meets the needs of the GP participants [20]. Similarly, the flexible nature of a community of practice enables clinicians to choose what topics they engage with and how [45]. Providing GPs with credible and relevant resources will equip GPs with practical tools for use in their clinical practice [28]. Together, the intervention components are likely to improve guideline concordance because they are based on social interactions, tailored to the needs of participating GPs, accompanied by credible and evidence-based advice from opinion leaders, and reinforced over a period of time [15, 16, 18].

The IMPRovE intervention has also been designed with acceptability and sustainability in mind. The intervention and its components were created in collaboration with a reference group of GPs and researchers, as well as an intervention advisory group that included GPs, mental health consumers, and representatives of workers’ compensation schemes. A steering group comprising members from three key clinical organisations (representing Australian GPs, psychologists and psychiatrists, respectively) as well as representatives of compensation agencies and a key non-government mental health organisation, *Beyond Blue*, also provided advice and guidance about the health system and policy context. Co-design with GPs who are the end-users of the intervention, and with policy makers who influence the compensation landscape and have the capacity to sustain the intervention into the future, should yield a more effective and sustainable intervention.

Finally, the IMPRovE trial has been designed using a practical approach to maximise feasibility. For instance, whilst guideline concordance would ideally be measured in routine clinical practice, GPs in our trial are likely to see a relatively small number of patients with work-related mental health conditions each year. Consequently, we have chosen to use virtual simulated patient scenarios as a practical yet rigorous approach to measuring our primary outcome of guideline concordance. We have also chosen this method of assessment due to the nature of the Guideline recommendations, which provide no specific outcomes that can be audited in a case note analysis. For instance, outcomes such as “consideration of social factors, or symptoms that may indicate comorbidities” may not be recorded in case notes, even if a GP does consider these factors in their clinical decision-making process. All secondary outcome measures are based on widely used pre-existing surveys or scales using established methodologies.

## Conclusions

Researchers, governments, and guideline developers invest significant time and funds to facilitate guideline implementation in primary care. The IMPRovE study will assess the effectiveness of a complex intervention on guideline implementation in the context of work-related mental health conditions. If this intervention proves to be effective and cost-effective, the IMPRovE study will chart a clear path to evidence-informed care for people with work-related mental health conditions.

## Supplementary Information

**Additional file 1:.** Mazza et al_Additional file a. TIDieRChecklistR0

**Additional file 2:.** Mazza_et al_Additional file b. CONSORTClusterChecklistR0

## Data Availability

Trial data can be obtained upon request from the corresponding author.

## Abbreviations

DMC: Data Monitoring Committee
GP: General practitioner
IMPRovE: Implementing work-related Mental health conditions in general PRacticE
ICC: Intraclass correlation coefficient
